# Comprehensive Transcriptome Analysis Reveals Sex-Specific Alternative Splicing Events in Zebrafish Gonads

**DOI:** 10.3390/life12091441

**Published:** 2022-09-16

**Authors:** Xing Lin, Fei Liu, Kaifeng Meng, Hairong Liu, Yuanli Zhao, Yuanyuan Chen, Wei Hu, Daji Luo

**Affiliations:** 1 State Key Laboratory of Freshwater Ecology and Biotechnology, Institute of Hydrobiology, The Innovative Academy of Seed Design, Hubei Hongshan Laboratory, University of Chinese Academy of Sciences, Wuhan 430072, China; 2 College of Advanced Agricultural Sciences, University of Chinese Academy of Sciences, Beijing 100049, China; 3 College of Fisheries, Huazhong Agricultural University, Wuhan 430070, China; 4 Southern Marine Science and Engineering Guangdong Laboratory (Zhanjiang), Zhanjiang 524088, China

**Keywords:** sex determination, gonadal differentiation, alternative splicing, RNA-seq, zebrafish

## Abstract

Alternative splicing is an important way of regulating gene functions in eukaryotes. Several key genes involved in sex determination and gonadal differentiation, such as *nr5a1* and *ddx4*, have sex-biased transcripts between males and females, suggesting a potential regulatory role of alternative splicing in gonads. Currently, the sex-specific alternative splicing events and genes have not been comprehensively studied at the genome-wide level in zebrafish. In this study, through global splicing analysis on three independent sets of RNA-seq data from matched zebrafish testes and ovaries, we identified 120 differentially spliced genes shared by the three datasets, most of which haven’t been reported before. Functional enrichment analysis showed that the GO terms of mRNA processing, mRNA metabolism and microtubule-based process were strongly enriched. The testis- and ovary-biased alternative splicing genes were identified, and part of them (*tp53bp1*, *tpx2*, *mapre1a*, *kif2c*, and *ncoa5*) were further validated by RT-PCR. Sequence characteristics analysis suggested that the lengths, GC contents, and splice site strengths of the alternative exons or introns may have different influences in different types of alternative splicing events. Interestingly, we identified an unexpected high proportion (over 70%) of non-frameshift exon-skipping events, suggesting that in these cases the two protein isoforms derived from alternative splicing may both have functions. Furthermore, as a representative example, we found that the alternative splicing of *ncoa5* causes the loss of a conserved RRM domain in the short transcript predominantly produced in testes. Our study discovers novel sex-specific alternative splicing events and genes with high reliabilities in zebrafish testes and ovaries, which would provide attractive targets for follow-up studies to reveal the biological significances of alternative splicing events and genes in sex determination and gonadal differentiation.

## 1. Introduction

Males and females show markedly sexual dimorphisms in many phenotypic traits, such as morphology, behavior, and physiology, although they share nearly identical genomes in most species [1,2,3]. Gonads are the primarily differential organs between the sexes and are frequently used to identify differentially expressed genes (DEGs) for sexual dimorphism studies [4]. Those sex-specific DEGs are supposed to be the intrinsic causes of sexually dimorphic characteristics [5]. Thousands of sex-biased genes have been identified from gonads in a diversity of species, including the fruit fly (*Drosophila melanogaster*) [6], mouse (*Mus musculus*) [7], and zebrafish (*Danio rerio*) [8]. Alternative splicing (AS) is a ubiquitous regulatory mechanism of gene expression, which expands the genome’s coding potential by producing multiple transcripts from a single gene [9]. These various transcripts may have different or even opposite functions [10,11]. Compared with the extensive studies of DEGs between ovaries and testes, the subject of sex-biased AS events and genes has rarely been reported at the genome-wide level, especially in fish.

Several key genes involved in sex determination and gonadal differentiation show differential splicing between male and female gonads [12]. For example, the sex-specific splicing of *nr5a1* genes on the Z and W chromosomes of the central bearded dragon (*Pogona vitticeps*) suppresses testis determination and contributes to sex determination [13]. In adult zebrafish, the long transcript of *ddx4* (also known as *vasa*) that contains the exon 4 is exclusively expressed in ovaries, while the short transcript lacking exon 4 is present only in testes [14]. These studies suggest that sex-specific alternative splicing may be another level of regulatory mechanism in sex determination and gonadal differentiation, in additional to the extensively studied sex-specific gene expression. Currently, how many sex-specific alternative splicing events exist in gonads and how these AS events affect the gene functions remain largely unclear. Thus, systematic identification of new differential AS events (DASEs) and differential AS genes (DASGs) between ovaries and testes through RNA-seq may uncover novel candidate genes and regulatory mechanisms in the related fields.

In recent years, zebrafish has emerged as an important model to study gonad development [15,16]. The zebrafish genome [17] has been sequenced and annotated to a very high standard, only comparable to the human (*Homo sapiens*) and mouse genomes, which greatly facilitates the identification of alternative splicing events based on deep RNA sequencing. Many studies have identified differentially expressed genes in zebrafish gonads through transcriptome analysis. However, a genome-wide identification of sex-specific alternative splicing events and genes in zebrafish ovaries and testes has not been reported yet.

In this study, we extracted three independent sets of RNA-seq data of the matched adult zebrafish ovaries and testes from the GEO database, to comprehensively identify the gonadal DASEs (gDASEs) and gonadal DASGs (gDASGs), globally. The sequence characteristics of these gDASEs, including the splice site scores, GC contents, intron and exon lengths, were analyzed to reveal the underlying factors that may affect the differential splicing between sexes. We also performed functional enrichment analysis to identify the main biological processes or pathways that these gDASGs may be involved in. Furthermore, the consequences of these gDASEs on protein structure and gene expression were analyzed to preliminarily elucidate their biological significances. Our work discovers novel sex-specific alternative splicing events and genes with high reliabilities, which would provide attractive targets for follow-up studies to reveal the biological roles of alternative splicing in sex determination and gonadal differentiation.

## 2. Materials and Methods

### 2.1. Fish Maintenance

The wild-type AB strain of zebrafish were maintained in a recirculation systems at 28.5 °C under an alternating 14 h/10 h light/dark cycle as described previously [18]. All experiments involving zebrafish were conducted following the Guide for the Care and Use of Laboratory Animals (https://www.ncbi.nlm.nih.gov/books/NBK54050/, accessed on 15 September 2021) and approved by the Ethics Committee of Institute of Hydrobiology, Chinese Academy of Sciences.

### 2.2. Collection of RNA-Seq Data

Series matrix files of the RNA-seq data: GSE142352, GSE142355, and GSE111882 [19,20,21] were downloaded from the public GEO database (http://www.ncbi.nlm.nih.gov/geo/, accessed on 24 September 2021). In each dataset, only the wild-type gonad samples were chosen for analysis. We obtained 2 female gonad samples and 2 male gonad samples for GSE142352, 2 female gonad samples and 2 male gonad samples for GSE142355, and 6 female gonad samples and 6 male gonad samples for GSE111882. In addition, PRJNA690124 [22] were downloaded from the public ENA database (https://www.ebi.ac.uk/ena/browser/, accessed on 7 October 2021), from which we obtained 3 brain samples, 3 gut samples, 3 heart samples, 3 kidney samples, 3 liver samples, 3 muscle samples and 3 spleen samples. Each sample is a pool of the corresponding tissues from female and male zebrafish.

### 2.3. Differential Gene Expression Analysis and Enrichment Analysis

The quality of the samples was assessed with FastQC (http://www.bioinformatics.babraham.ac.uk/projects/fastqc/, accessed on 15 October 2021). Sequences were then trimmed using Trimmomatic software v0.36 (Bjoern Usadel, Düsseldorf, Germany), accessed on 15 October 2021 [23]. Each sample was aligned against the reference genome (GRCz11) using STAR software v2.7.5c (Thomas R Gingeras, NY, USA), accessed on 15 October 2021 [24]. Pure read counts were extracted from the alignment files using featureCounts v2.0.1 (Wei Shi, Parkville, Australia), accessed on 15 October 2021 [25]. Analysis and visualization of RNA-seq data were performed in the R statistical environment v4.0.2(R core team, Vienna, Austria), accessed on 15 October 2021. Normalization and differential expression analysis were performed by R/Bioconductor package DESeq2 [26]. Genes were considered differentially expressed when the adjusted *p*-value ≤ 0.01 and −2 < log2foldchange > 2. Then DEGs were enriched and analyzed with Gene Ontology (GO) term [27] using clusterProfiler v 3.12.0 (Guangchuang Yu, Guangzhou, China) [28], *p*-value < 0.05 set as the cutoff criterion.

### 2.4. Differential AS Analysis and Validation

Sequencing reads were mapped to the assembly transcript using the STAR aligner v2.7.5c in the two-pass mapping mode. The DASEs were identified based on assembled transcript sequences obtained from the RNA-Seq sequencing using rMATS with default parameters [29]. Integrative Genomics Viewer v2.3.91 (Jill P Mesirov, Cambridge, MA, USA), accessed on 5 May 2022, was used to visualize RNA-Seq tracks [30]. RT-PCR was conducted for seven selected genes, *cenpe*, *kif2c*, *mapre1a*, *ncoa5*, *tpx2*, and *wdr62*, to validate the AS events identified by RNA-seq. Total RNA samples were extracted separately from the gonads, gills, eyes, brains, hearts, livers, spleens, kidneys, guts and muscles from the adult at 6 mpf (month post fertilization, mpf) male and female zebrafish using the Trizol reagent (Invitrogen, Waltham, MA, USA), according to the manufacturer’s protocol. RNA concentration and purity were measured by a Nanodrop ND-2000 spectrophotometer (Thermo, Waltham, MA, USA). RNA integrity was assessed using agarose gel electrophoresis. The PrimeScript II 1stStrand cDNA Synthesis Kit (TaKaRa, Kusatsu, Japan) and SYBR Premix Ex Taq II (TaKaRa, Kusatsu, Japan) were used for reverse transcription reaction and the PCR assay. Specific primers (Table 1) for the selected genes were designed using NCBI Primer-BLAST (NCBI, Bethesda, MD, USA) according to the homologous flanking sequences or specific splicing of exons for all potential AS transcript isoforms. The PCR amplification cycle was as follows: 35 cycles at 95 °C for 15 s, 58 °C for 15 s, and 72 °C for 15 s followed by 72 °C for 3 min. PCR products were examined and isolated on a 1% agarose gel.

### 2.5. Splice Site Scoring, GC Content and Length Calculating

Log-odds scores for splice sites were computed using maximum entropy (MaxEnt) method by the website tool [31] (http://hollywood.mit.edu/burgelab/software.html, accessed on 15 October 2021). According to the exons component of splicing events, the start and end positions of the alternative fragment were confirmed and further used to get the limits of splice site regions in different splice types separately. Sequences of splice site regions were from genome sequence (GRCz11) and were used to calculate MaxEnt score for 3′ splice site (3′ss) and 5′ splice site (5′ss), respectively. Guanine and cytosine (GC) content and length of the alternative fragments were calculated according to their start and end positions using bedtools software v2.29.2 (Ira M Hall, Charlottesville, USA), accessed on 1 May 2022 [32].

### 2.6. Protein–Protein Interaction (PPI)

Candidate target genes and other gDASGs were imported into the STRING [33], a database of known and predicted PPIs. We selected “*Danio rerio*” and a medium confidence score with a correlation degree ≥0.400 as the cut-off value.

### 2.7. Three-Dimensional Protein Structure Prediction and Analysis

The three-dimensional structures of the putative proteins encoded by the original and differentially spliced transcripts of *ncoa5* were predicted with AlphaFold [34], respectively. The PyMOL Molecular Graphics System (Schrödinger, New York, USA) was utilized for the visualization of the predicted structures (http://www.pymol.org, accessed on 10 May 2022).

### 2.8. Statistical Analysis and Data Visualization

Statistical analyses were performed using the R software (version 4.0.2, R core team, Vienna, Austria), Python (version 3.9, Python Software Foundation, Wilmington, NC, USA) and Graphpad Prism (version 9.0.0, San Diego, CA, USA). Data analysis and visualization tools in R software included the ggplot2 and pheatmap packages.

## 3. Results

### 3.1. Identification of gDEGs and gDASGs between Zebrafish Ovaries and Testes Based on RNA-Seq Data

**Figure 1:**
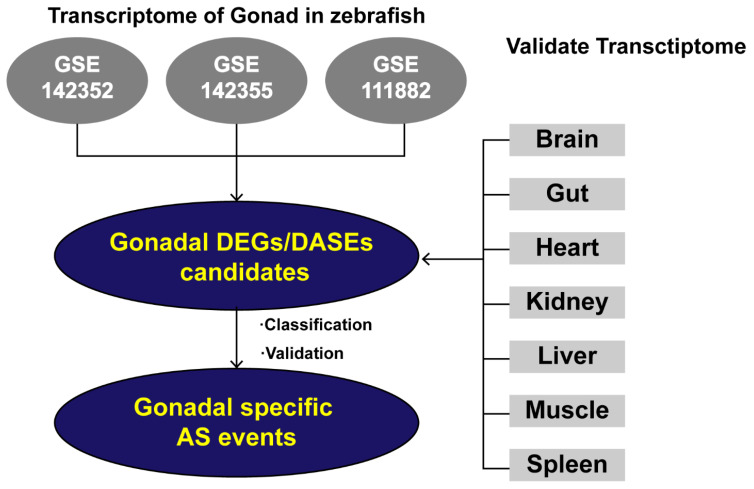
Analytical workflow. Three independent sets of RNA-seq data (GSE142352, GSE142355, GSE111882) from matched zebrafish testes and ovaries were used to conduct genome-wide analysis of gonadal expression levels and AS events in female and male zebrafish. The DEGs and DASGs shared by the three data sets are referred to as gDEGs and gDASGs. Following this, the gDASGs were compared with RNA-Seq data (PRJNA690124) from brain, gut, heart, kidney, liver, muscle, spleen to obtain gonadal specific gDASGs.

**Figure 2:**
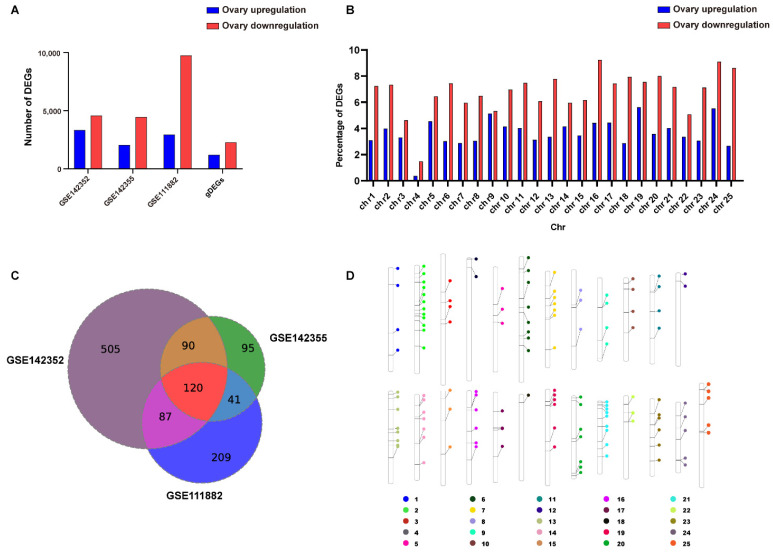
Number of DEGs and DASGs in zebrafish and chromosome distribution profile for clusters of gDEGs and gDASGs (**A**) Bar graph representing the statistics of DEGs highly expressed in ovary and testis respectively. (**B**) Relative chromosome distributions in percentages of gDEGs in testis and ovary. (**C**) Venn diagram of DASGs at different datasets. (**D**) Distribution of gDASGs on chromosomes.

Three independent sets of RNA-seq data from matched zebrafish testes and ovaries were used to identify gDEGs and gDASEs at the genome-wide level. The candidate gDASEs were further retrieved in the RNA-Seq data from other seven tissues (brain, gut, heart, kidney, liver, muscle and spleen) of zebrafish, to see if these gDASEs were only present or enriched in gonads. Furthermore, the gDASEs were classified into different alternative splicing types and validated by RT-PCR (Figure 1). As a result, 7913, 6516, and 12714 DEGs were identified (Table S1). Among them, 3337, 2055, and 2947 genes were upregulated in zebrafish ovaries, while 4576, 4461, 9767 were upregulated in zebrafish testes in GSE142352, GSE142355, and GSE111882, respectively (Figure 2A).

A total of 3482 gDEGs were shared by the three datasets, including 2284 genes upregulated in testes and 1198 genes upregulated in ovaries (Figure 2A), implying that adult male zebrafish had higher levels of active gene expression. To explore the possible sex chromosomes of zebrafish, we analyzed the distribution of these gDEGs in the zebrafish genome and found that a slightly higher proportion of testis-upregulated gDEGs were located on chromosome 4 (Figure 2B, Table S2), indicating that chromosome 4 may be the potential sex chromosome of zebrafish. To understand the potential pathways leading to gender differences, we also performed functional enrichment analysis. GO term functional enrichment analysis based on the identified gDEGs revealed that the plasma membrane bounded cell projection assembly, cell projection assembly, axoneme assembly, and cilium assembly GO terms were significantly enriched in zebrafish testes, whereas oogenesis, female gamete generation, egg coat formation and ncRNA processing GO terms were enriched in zebrafish ovaries (Figure S1, Table S3).

In addition to the gDEGs, 802, 346, and 457 DASGs were identified in GSE142352, GSE142355, and GSE111882, respectively (Figure 2C). Among them, 120 gDASGs with 150 gDASEs were shared by the three datasets (Figure 2C, Table S4). To explore potential sex chromosomes of zebrafish, we explore the distribution differences of gDASGs on chromosomes. Results shows that the chromosome 2 contains the most gDASGs, whereas the chromosome 18 contains the least gDASGs, indicating that AS events in zebrafish gonads were not evenly distributed between chromosomes (Figure 2D). Besides, we also performed functional enrichment analysis. GO terms enrichment analysis based on each group of DASGs identified almost the same enriched GO terms, including mRNA processing, mRNA metabolic process, regulation of mRNA metabolic process and RNA splicing and microtubule-based process. However, genes that showed differential AS in testes were significantly enriched in mRNA processing, negative regulation of cell cycle GO terms, and genes that showed differential AS in ovaries were significantly enriched in RNA splicing, mRNA processing, microtubule cytoskeleton organization GO terms (Figure S2).

### 3.2. Classification and Validation of the Gonad-Specific AS Events

**Figure 3:**
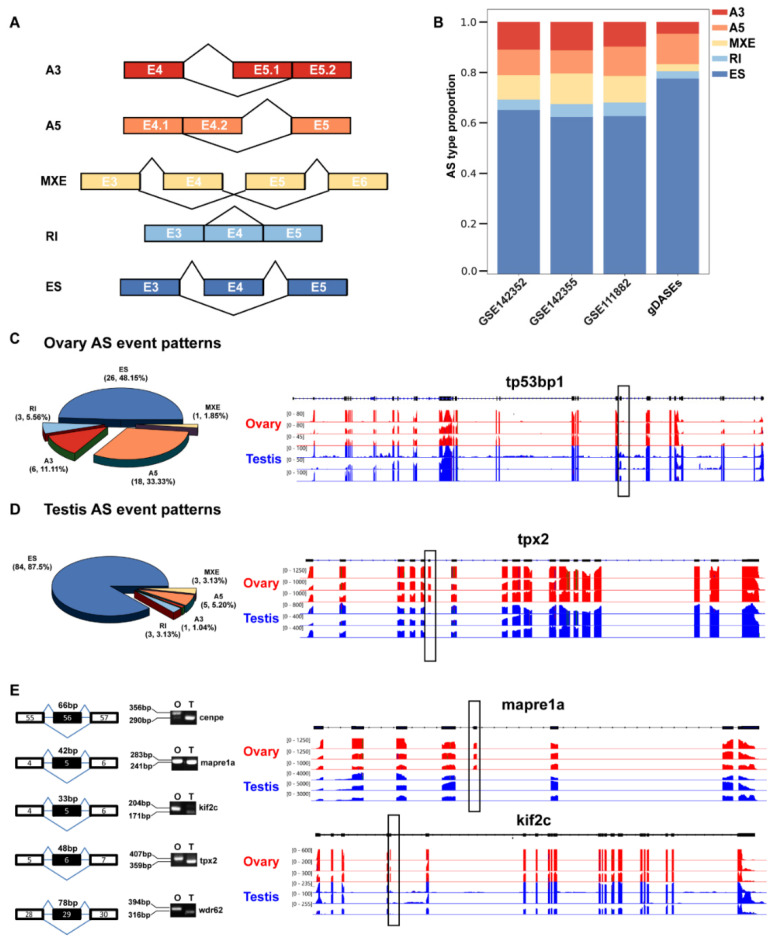
Distribution of AS types and in frame ratio in male and female zebrafish gonad. (**A**) Splice model for splice types detected in the rMATS projects. Exon number was illustrated in the box. (**B**) Proportion of the identified DASEs. ES, exon skipping; A5SS, alternative 5′ splice site; A3SS, alternative 3′ splice site; MXE, mutually exclusive exon; RI, retained intron; (**C**) Proportion of the gDASEs in ovary and case. (**D**) Proportion of the gDASEs in testis and case. (**E**) The differentially spliced transcripts of *mapre1a*, *tpx2*, *cenpe*, *kif2c* and *wdr62* were validated by RT-PCR using cDNA samples from adult (6 mpf) zebrafish gonads (O for ovaries and T for testes). The RNA-seq tracks of *tp53bp1*, *tpx2*, *mapre1a*, and *kif2c* are shown in the right panel. The differentially spliced exons between ovaries and testes are indicated with boxes.

To study the changes of DASEs between zebrafish gonads, the mRNA splicing patterns in the testes were compared with those in the ovary. The gDASEs were classified into five groups: alternative 3′ splice site (A3SS), alternative 5′ splice site (A5SS), exon skipping (ES), intron retention (IR), and mutually exclusive exons (MXE) (Figure 3A). Our results revealed that the ES events were the most common AS events, accounting for 65.1%, 76.3%, and 62.6% in the three datasets, respectively (Figure 3B). We also found that ES events accounted for 48.15% of the DASEs in the ovary and 87.5% of the DASEs in the testis of zebrafish. The percentages of the other four types of AS events in the ovary were 11.11%, 33.33%, 5.56% and 1.85% for A3SS, A5SS, RI and MXE, respectively. In the testis, the corresponding percentages were 1.04%, 5.20%, 3.13% and 3.13% for A3SS, A5SS, RI and MXE, respectively (Figure 3C,D).

To prove the accuracy of the results, two representative gDASEs in *tpx2* and *tp53bp1* genes were selected and visualized by IGV. *Tp53bp1* is required for the maturation of oocytes in mouse [35]. In female zebrafish gonads, the exon 20 of *tp53bp1* was largely skipped (Figure 3C). *Tpx2* is a multifunctional protein involved in the mitotic/meiotic activities [36] and spermatogenesis [37]. The exon 6 of *tpx2* was almost completely skipped in the male gonads of zebrafish (Figure 3D). Furthermore, to validate the accuracy of the gDASGs found in the gonads of male and female zebrafish, 5 genes (*cenpe*, *kif2c*, *mapre1a*, *tpx2* and *wdr62*) were chosen for RT-PCR verification (Figure 3E).

### 3.3. Sequence Characteristics of the AS Exons and Introns in Ovaries and Testes

**Figure 4:**
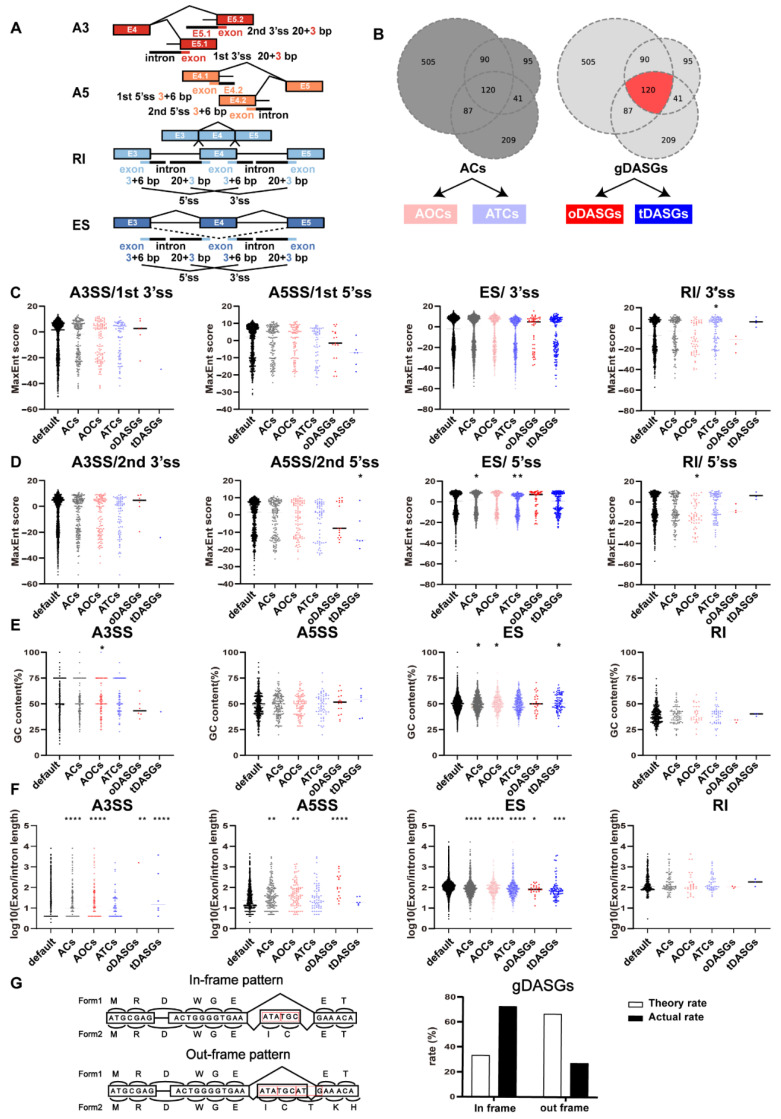
Sequence characteristics of the AS exons and introns in ovaries and testes. (**A**) Models of splicing related SNPs in different regions for A3SS, A5SS, ES and RI separately. SNPs were classified by position as four mutually exclusive regions, including splice (3′ss and 5′ss), exon (alternative fragment), gene (the splicing gene) and chromosome (out of the splicing gene). (**B**) model of different groups of DASGs (ACs:All Candidates, AOCs: All Ovary Candidates, ATCs: All Testis Candidates, oDASGs: Ovary specific gDASGs, tDASGs: Testis specific gDASGs). (**C**,**D**) MaxEnt score of 5′ss and 3′ss, (**E**) GC content and (**F**) length of alternative fragment in splice type A3SS, A5SS, ES and RI, separately.(**G**) model of frame-preserving or frame-switching and theory rate and actual rate of gDASGs. Exon length determines whether an alternatively spliced single-exon skip is frame-preserving or frame-switching. We define an alternatively spliced exon as frame-preserving if its length is an exact multiple of 3 nt, as its alternative splicing will not alter the protein reading frame of subsequence exons (top). *, *p* < 0.05; **, *p* < 0.01; ***, *p* < 0.001; ****, *p* < 0.0001.

To delineate splicing features of gDASEs in ovary and testis, the sequence characteristics of the exons or introns involved in the gDASEs were analyzed. Splice site score, GC content, and alternative fragment length were calculated separately for each DASEs to distinguish splicing features in different groups (ACs: All Candidates, AOCs: All Ovary Candidates, ATCs: All Testis Candidates, oDASGs: Ovary specific gDASGs, tDASGs: Testis specific gDASGs) (Figure 4B). Splice site strengths were calculated using MaxEntScan [31], where the input for the 5′ model was the sequence 6 bp into the intron and 3 bp into the exon, and where the input for the 3′ model was the sequence 20 bp into the intron and 3 bp into the exon (Figure 4A).

In our study, gDASGs with ES were notably characterized by having shorter alternative exons and higher GC contents (Figure 4E,F; Tables S6 and S7). Stronger 5’ss were more common in testis specific gDASGs with ES compared with ovary specific DASEs with ES, which may cause the ovaries to have upregulated PSI. However, no discernible alterations in GC content or alternate intron length were present in gDASGs with RI. Contrarily, weaker 5’ss were substantially associated with ovary specific gDASGs with RI, whereas stronger 3’ss were associated with testis specific gDASGs with RI (Figure 4C,D). For A3SS and A5ss, ovary specific gDASGs with higher GC content and longer length were discovered (Figure 4E,F; Table S5).

To explore the effect of sequence length change on protein structure, we analyzed the frame-preserving ratio. Interestingly, we discovered that the proportion of non-frameshift gDASEs is 72.6%, demonstrating that gDASGs are more likely to retain the open reading frames (Figure 4G). To verify whether this phenomenon is unique in gonads, RNA-Seq data of zebrafish liver, gut, kidney, spleen, brain, heart and muscle (PRJNA690124) were supplemented for AS analysis. Our results revealed that the proportions of non-frameshift AS events are 81% (brain vs. ovary), 83% (gut vs. ovary), 81% (heart vs. ovary), 82% (kidney vs. ovary), 83% (liver vs. ovary), 83% (muscle vs. ovary), 79% (spleen vs. ovary), 78% (brain vs. testis), 76% (gut vs. testis), 76% (heart vs. testis), 76% (kidney vs. testis), 76% (liver vs. testis), 77% (muscle vs. testis), and 75% (spleen vs. testis), respectively (Figure S3). The non-frameshift alternate exons have a higher delta PSI (Figure S3), indicating that this phenomenon is common.

### 3.4. Ovary-Specific Constituent Exons Are Associated with Microtubule Function

**Figure 5:**
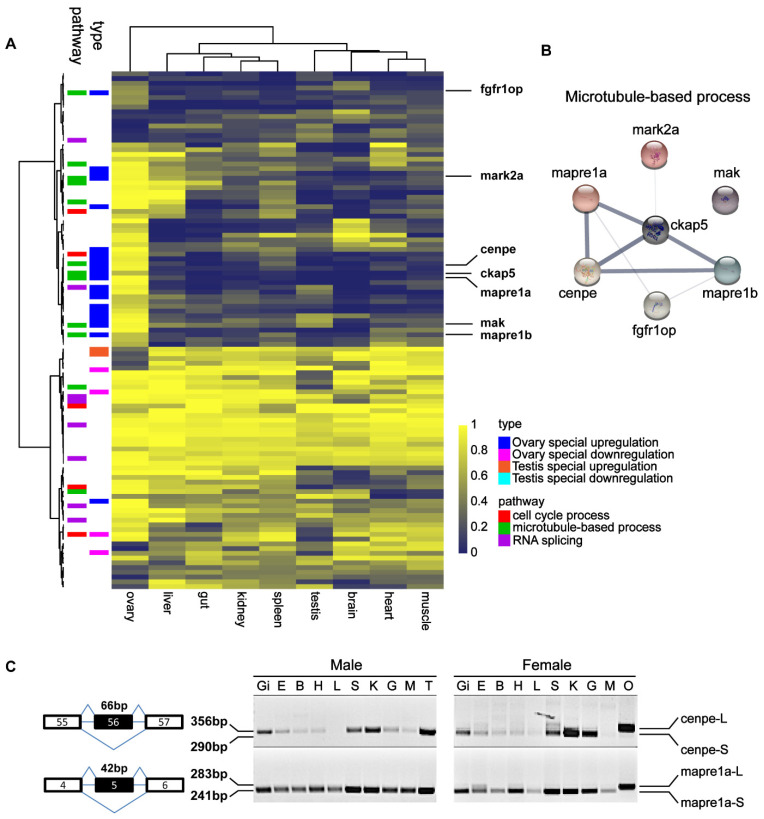
Identification of testis- and ovary-specific AS genes using multi-tissue RNA-Seq data. (**A**) Heatmap of PSI value of ovary, liver, gut, kidney, spleen, testis, brain, heart and muscle. (**B**) Protein-protein interaction network. (**C**) Differentially expressed splicing transcripts of *cenpe* and *mapre1a* were validated by RT-PCR in gonads and other tissues from adult (6 mpf) male and female zebrafish. The DNA fragments were sequenced and shown in Figure S6. ‘Gi’: gill; ‘E’: eye; ‘B’: brain; ‘H’: heart; ‘L’: liver; ‘S’: spleen; ‘K’: kidney; ‘G’: gut; ‘M’: muscle; ‘T’: testis; and ‘O’: ovary.

To obtain ovary- and testis-specific DASGs, the multi-tissue RNA-Seq data was used for integrated analysis with previous gonad RNA-Seq data (GSE142352, GSE142355, and GSE111882) to identify gonadal specific DASEs that might be regulated by the splicing code in germ cells. A list of potential testis- or ovary-specific exons was obtained and the PPI network’s module analysis was performed (Table S8, Figures S4 and S5). Surprisingly, most microtubule-based process genes, such as *cenpe*, *ckap5*, *fgfr1op*, *mak*, *mapre1a*, *mapre1b*, and *mark2a,* have ovary-specific exons, and the most common type is ovary-specific exon retention (Figure 5A). The PPI network’s module analysis suggested that the microtubule-based process may be involved in the maintenance of ovary function. (Figure 5B). To demonstrate the accuracy of the ovary- and testis-specific DASGs, RT-PCR and Sanger sequencing were performed to validate the gDASEs in *mapre1a* and *cenpe* genes in the gonads and other nine tissues from the adult male and female zebrafish (Figure 5C and Figure S6).

### 3.5. Determining the Possible Consequence of a Sex-Specific AS Event on the Function of ncoa5 Gene by Conservation and 3D Structure Analysis

**Figure 6:**
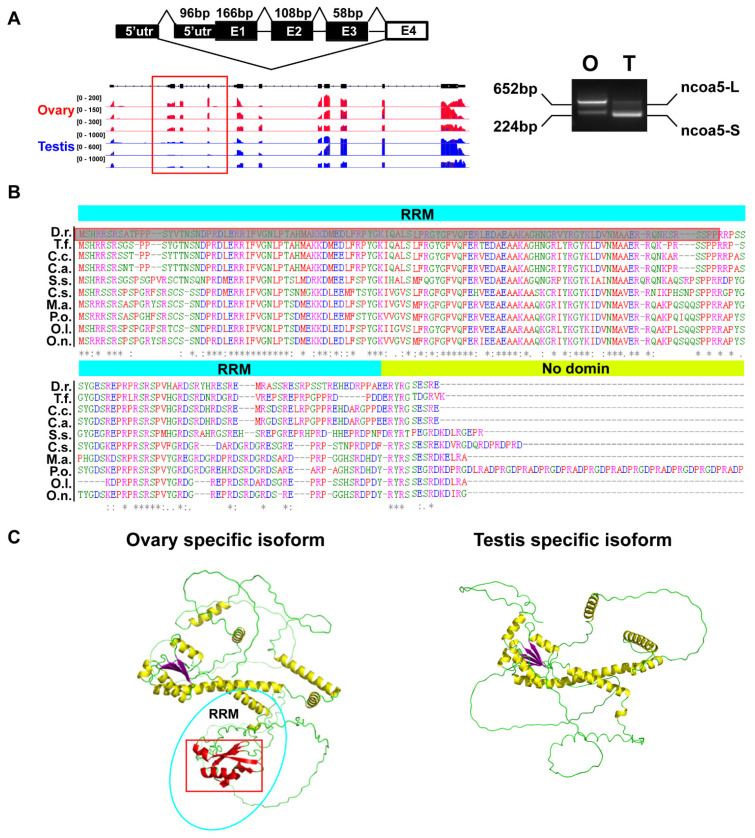
Cases for the biological significances of the sex specific AS events. (**A**) The differentially spliced transcripts of *ncoa5* were validated in adult (6 mpf) zebrafish gonads (O for ovaries and T for testes) by RT-PCR. (**B**) Multiple protein sequence alignment of RRM1 from *Tachysurus fulvidraco* (T.f.; XP_027002460.1), *Danio rerio* (D.r.; XP_005166217.1), *Cyprinus carpio* (C.c.; XP_018925131.1), *Carassius auratus* (C.a.; XP_026121940.1), *Salmo salar* (S.s.; NP_001133374.1), *Cynoglossus semilaevis* (C.s.; XP_024915677.1), *Oryzias latipes* (O.l.; XP_023810720.1), *Paralichthys olivaceus* (P.o.; XP_019952347.1), *Oreochromis niloticus* (O.n.; XP_019214575.1) and *Monopterus albus* (M.a.;XP_020442329.1) obtained with Clustal Omega, the skip region is marked by red box (grey bottom). The asterisk (*) indicates the identical amino acid residue. (**C**) 3D protein structure modeling of *ncoa5-S* and *ncoa5-L*, with the RRM domain represented by a blue ellipse and the skip region designated by a red box, both of which contain sheet and helical structures.

We investigated the dynamics of transcription start site usage in ovary and testis. A representative example of our data, *ncoa5*, is consistent with the possibility that the gonad of adult male zebrafish mainly utilize proximal transcription start site to express a short mRNA *ncoa5-S*, whereas the gonad of female zebrafish switch to distal transcription start site to express a longer mRNA *ncoa5-L* (Figure 6A), and RT-PCR was used to validate the down-regulated alternative use of *ncoa5* exon in male zebrafish gonads. Furthermore, despite the fact that the length of other exons in *ncoa5* varied widely across various fish genomes, the sequence and length of this RRM domain were substantially conserved (Figure 6B), implying that this sequence may have an important function in fish and plays an important role in zebrafish gonads. Figure 6C depicts the 3D protein structure modeling of *ncoa5-S* and *ncoa5-L*, with the RRM domain represented by a blue ellipse and the skip region designated by a red box, both of which contain sheet and helical structures.

## 4. Discussion

AS of pre-mRNA is one of the fundamental mechanisms for the regulation of gene expression in higher eukaryotes [38]. AS is observed in about 92–94% of genes in human [11] and plays a key role in sex determination in mammals [12]. Meanwhile, a large number of gonadal-associated AS genes have been discovered in mice [9], Xiang pig [39] and fruit fly [40] using deep RNA-Seq. However, sex-specific AS events have not been comprehensively studied at the genome-wide level in zebrafish, a popular model organism in life sciences. Our study discovers novel sex-specific alternative splicing events and genes with high reliabilities in zebrafish testes and ovaries, which would provide attractive targets for follow-up studies to reveal the biological significances of alternative splicing events and genes in sex determination and gonadal differentiation.

Because the results of individual experiments can be noisy, it is critical to look for findings that are supported by multiple pieces of evidence to increase the signal and reduce the fraction of false-positive findings. In short, we found 3480 shared gDEGs and 120 shared gDASGs (Figure 2A,C). Similarly to the findings in mice [9], we discovered more upregulated genes in testis than in ovary, which is consistent with sperm being continuously produced in very large numbers within the testes and periodic release in this species [8]. Meanwhile, the GO annotation analysis clearly revealed that a lot of ovary-upregulated gDEGs were involved in oogenesis, female gamete generation and egg coat formation, which indicate that oogenesis and female gamete generation is an important function of the ovary. Importantly, whether analyzing a single piece of data or multiple sets of data, DEGs and DASGs were significantly enriched in plasma membrane bounded cell projection assembly, cell projection assembly, axoneme assembly, cilium assembly, mRNA processing and mRNA metabolic process GO terms, indicating that the microtubule function plays an important role in maintaining sexual dimorphism (Figures S1 and S2). However, in the current study, we discover relatively little overlap between gDASGs and gDEGs (2 testis-upregulated genes and 2 ovary-upregulated genes) (Table S9), implying that the gene splicing response to maintaining sexual dimorphism is functionally distinct from the transcriptional response (Figure S7). Our findings support the notion that gene-level measurements can miss potentially important isoform-level genetic changes [8].

Subsequently, we analyze the frame-preserving ratio of 120 shared gDASGs (Figure 4G). To the best of our knowledge, the expected probability of a given exon being frame-preserving (an exact multiple of 3 nt in length) by pure random chance is 33% [41]. However, we discovered that the probability of frame-preserving is 72.6% and that delta PSI is negatively correlated with the frame-preservation ratio; this finding is consistent with previous research studies [11,41]. Furthermore, Sorek [42] discovered that 77% of conserved interspecies AS exons did not change the reading frame and that orthologous exons have functional importance. Besides, skipped exons with lengths divisible by three can also play a significant role. Although these exons does not completely eliminate protein function [43], it may be actively used to regulate protein activity or localization [44].For example, two AS donor sites at the end of exon 9 of *Wt1* resulted in the insertion or omission of three amino acids (KTS) between zinc fingers 3 and 4 (designated as +KTS and −KTS isoforms), without interrupting the open reading frame [45]. Interestingly, KTS splice variants of *Wt1* have distinct functions in mouse [46] and only the −KTS form of *Wt1* can bind to and transactivate the *Sf1* promoter [47].

However, how the prevailing conditions dictate which isoform is expressed and what biological factors might influence the regulation of this process remain areas requiring further exploration [48]. We have previously found that the proportion of frame-preserving is higher than expected and delta PSI is negatively correlated with the frame-preservation ratio (Figure S3). Furthermore, accurate pre-mRNA splicing requires regular splice sites and cis-acting elements [49], indicating that sequence characteristics affect the efficiency of splicing. Here, we define non-significant AS events (0.001 < delta PSI < 0.1) as a control group and significant AS events (delta PSI > 0.1) divided into different groups (ACs: All Candidates, AOCs: All Ovary Candidates, ATCs: All Testis Candidates, oDASGs: Ovary specific gDASGs, tDASGs: Testis specific gDASGs). Because GC content influences the characteristics involved in splice-site recognition, it is possible that GC content may also have a direct impact on splice-site recognition [50]. We discovered that the GC content is positively correlated with the reliability level of alternative exons in our study (Figure 4E). High GC content appears to impair the splicing mechanism’s ability to recognize these exons, resulting in exon skipping (tDASGs vs. control and oDASGs vs. control). In contrast, A3SS, A5SS, and RI were characterized by consistent GC content, and the GC content of RI was far less than other types (Figure 4E). In addition, exon/intron length also influences splicing efficiency as reported previously [51]. In our study, exon length is negatively correlated with the reliability level of alternative exons in ES, but positively correlated with the reliability level of alternative exons in A5SS and A3SS. However, no intron changes were discovered (Figure 4E,F), which contradicts previous findings on mammals that introns with high GC content and short intron lengths are important and common features of intron retention [52].

The splicing machinery can be controlled at a number of different levels, and is frequently tissue-specific [53]. Previous studies have collected 102 testis-specific exons in mice [54]. In humans, the ovary and testis each accounted for at least 4% of the observed tissue-specific forms [55]. In our study, we supplemented RNA-Seq data of zebrafish liver, gut, kidney, spleen, brain, heart and muscle in order to obtain zebrafish gonad specific transcripts that are identical to those found in mammals. Different actions of splicing regulators expressed in different cell types may result in the generation of tissue-specific exons [56,57] and tissue-specific exons will affect the function of the corresponding tissue. For example, a testis-specific exon between exons 6 and 7 of the human *TLE4* gene inserts an additional 39 nucleotides encoding 13 amino acids into the *TLE4* protein, resulting in regulation of signaling pathways in the testis [54]. Previous research discovered the single exon deletion variant D6 as well as the double deletion variants D4/6 and D6/7 of the *Esr* gene in cat testicular tissue exclusively, specifically regulating the cellular susceptibility to hormonal stimuli within the gonads [58]. Of the nine tissues examined in our study, the *ncoa5* gene only displayed exon retention in the ovary, which encodes a coregulator for nuclear receptor subfamily 1 group D member 2 and estrogen receptors 1 and 2 [59], positively regulating the expression of aromatase. Gonad of adult male zebrafish mainly utilize proximal transcription start site to express a short mRNA *ncoa5-S*, whereas gonad of female zebrafish switch to distal transcription start site to express a longer mRNA *ncoa5-L* (Figure 6A). Indeed, *ncoa5* has one RNA recognition motif (RRM) domain, but due to the change from distal to proximal transcription start site, *ncoa5-S* encodes an N-terminal truncated version with loss of this RRM domains. The RRM functional domain, on the other hand, has been linked to a variety of important functions, including spermatogenesis and apoptosis.

Surprisingly, the majority of ovary specific DASGs are linked with microtubule activity in the present study (Figure 5A). The microtubule is involved in the regulation of cell cycle progression, mitosis and meiosis [60,61], and microtubule function research is critical for understanding the molecular mechanisms behind several developmental processes that occur throughout zebrafish oogenesis [62]. We used the STRING algorithm to identify putative interactions. Basically, one interactive network was predicted consisting of *cenpe*, *ckap5*, *fgfr1op*, *mak*, *mapre1a*, *mapre1b* and *mark2a* (Figure 5B), and only one gene was excluded from the interaction network, indicating that these ovary-specific gDASGs have close functional relationships, and the unique expression of AS exons may affect oogenesis, mitosis, and meiosis. Overall, our findings provide new insights into the role of AS in oogenesis and spermatogenesis.

## 5. Conclusions

Alternative splicing may play an important role in sex determination and gonadal differentiation. The alternative splicing events in fish gonads were usually concentrated on few genes for each separate study and rarely studied at the genome-wide level in the past. Therefore, this study performed global splicing analysis on several independent RNA-seq datasets of adult zebrafish testes and ovaries, intending to depict a landscape of the sex-specific alternative splicing events in zebrafish gonads and determine the fundamental features of these events. This study provides novel sex-specific alternative splicing events and genes with high reliabilities. Functional enrichment analysis suggested that mRNA processing, mRNA metabolism and microtubule-based process were more likely regulated by these sex-specific alternative splicing events. The sequence characteristics of the alternative exons or introns, including the lengths, GC content and splice site strengths, were determined and analyzed. In addition, we found an unexpected high proportion of non-frameshift exon-skipping events, suggesting that in these cases the two protein isoforms derived from alternative splicing may both have functions. As a representative case, the consequence of the sex-specific alternative splicing event in *ncoa5* were determined by conservation analysis and 3D structure prediction. Overall, our work has proposed attractive targets for revealing the biological roles of alternative splicing in fish sex determination and gonadal differentiation in the future.

## Figures and Tables

**Table 1:** List of primers. Specific primers for the selected genes were designed using NCBI Primer-BLAST (NCBI, USA) according to the homologous flanking sequences or specific splicing of exons for all potential AS transcript isoforms.

Name of Primer (RT-PCR)	Sequence (5′ to 3′ Direction)
*Cenpe*-F	GACCGAAGTCAACTCACCCA
*Cenpe*-R	ACTGTAGTACTGACGCGCTG
*kif2c*-F	CACCCCTATTCCGACACCAA
*kif2c*-R	GAAGATGCCGAGACCACAGG
*mapre1a*-F	AGCTTGTGAAGGGCAAGTTTC
*mapre1a*-R	GTTGCCGCAGGAGATGTTTTT
*ncoa5*-F	CGCACTTCCGCACAGGATTA
*ncoa5*-R	CTCTCGACTTTCACGGTGGT
*tpx2*-F	AGACAAGAGCCGACAAGCTC
*tpx2*-R	GCCTTTAGTCGGACACTGCT
*wdr62*-F	GTTTCCGCAGTGCTGCTTGTC
*wdr62*-R	TCCACGGAGTTGCTTTGGTT

## Data Availability

Access to the data presented in this study is available in the Materials and Methods section and in the Supplementary Materials section.

## Supplementary Materials

The following supporting information can be downloaded at: https://www.mdpi.com/article/10.3390/life12091441/s1.
