# Supplementary material for: Comprehensive Transcriptome Analysis Reveals Sex-Specific Alternative Splicing Events in Zebrafish Gonads
Source: Life (Basel). 2022 Sep 16;12(9):1441. doi: 10.3390/life12091441 (PMC9501657; doi:10.3390/life12091441)
Supplement: Supplementary file 1 [file life-12-01441-s001.zip › Supplementary Figures R1.pdf]

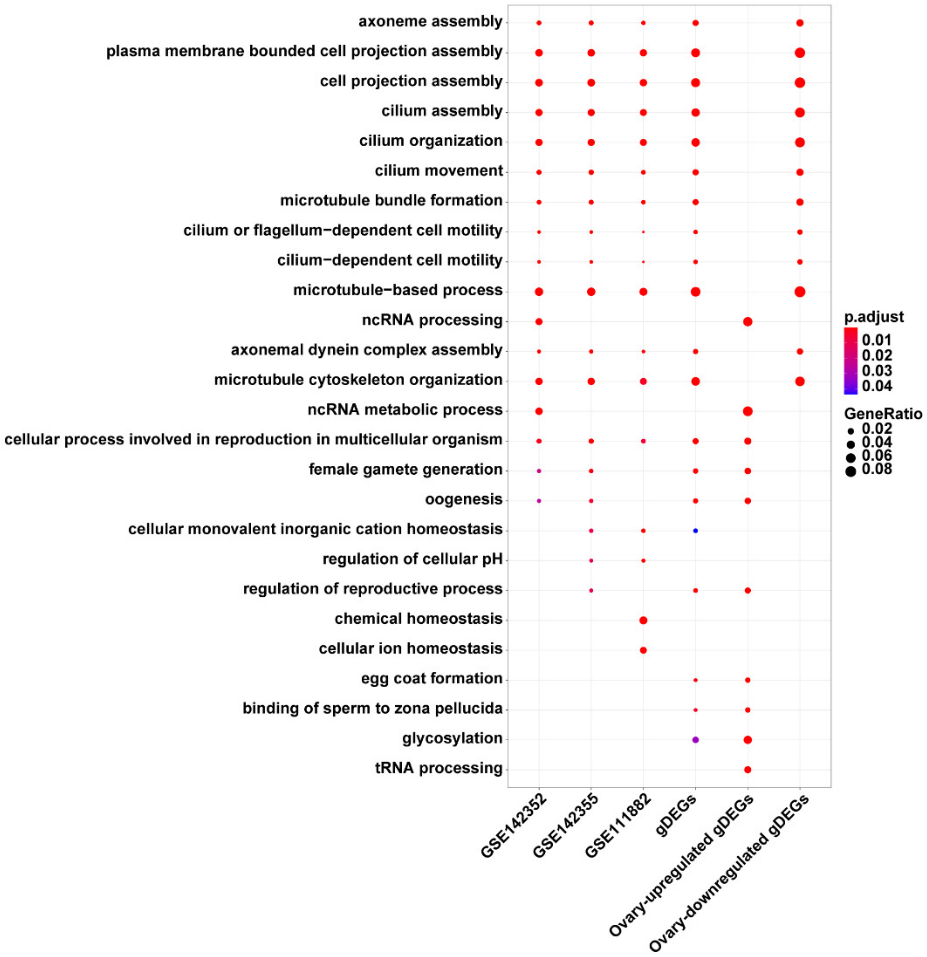

Figure S1: GO annotations of different groups (GSE142352, GSE142355,GSE111882 ,gDEGs, Ovary-upregulated gDEGs, Ovary-downregulated gDEGs).

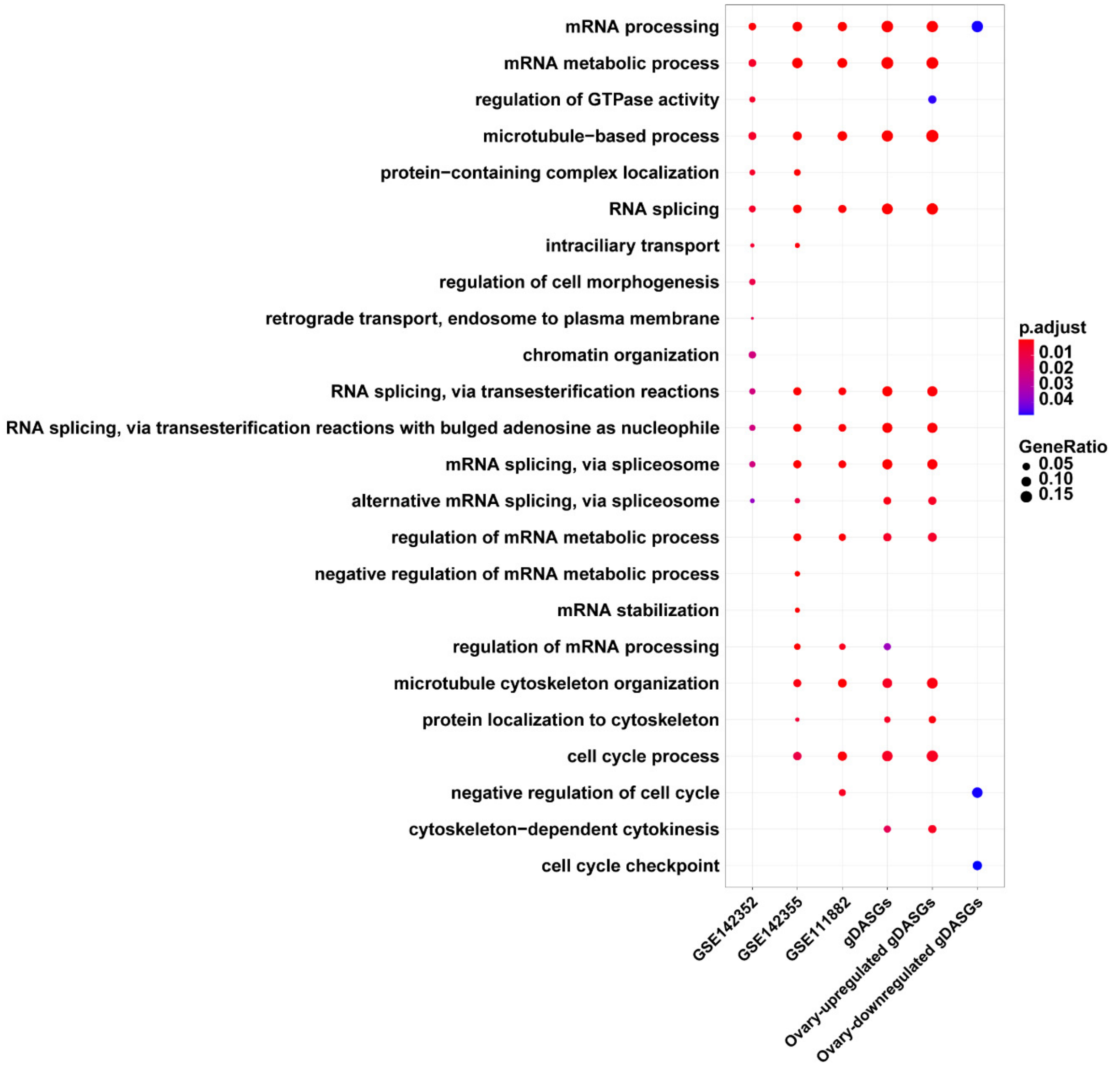

Figure S2: GO annotations of different groups (GSE142352, GSE142355,GSE111882 ,gDASGs, Ovary-upregulated gDASGs, Ovary-downregulated gDASGs).

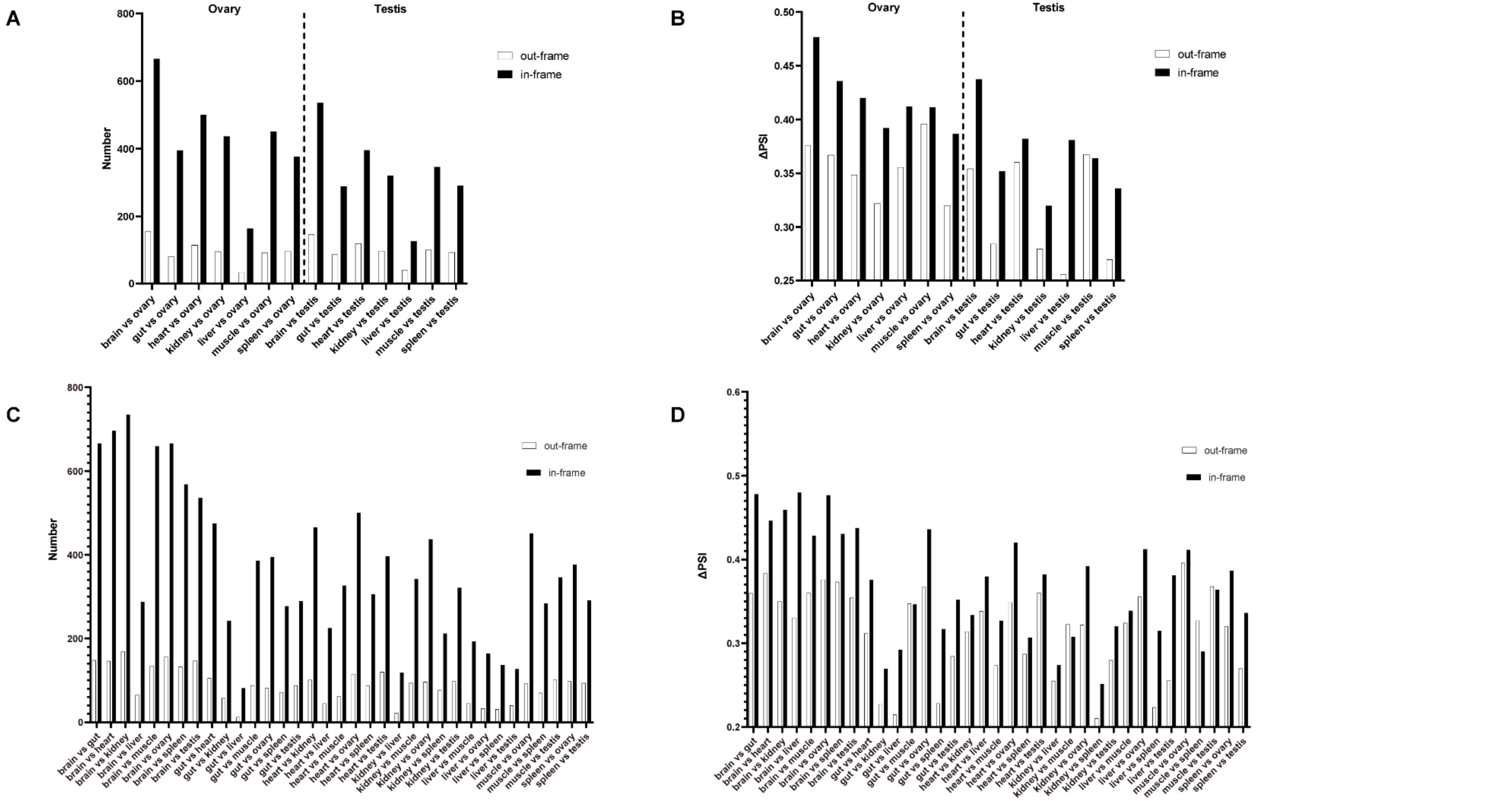

Figure S3: The AS analysis was carried out for nine tissues. (A) Ovary and testis frame-preservation gene numbers and (B) its corresponding delta-PSI compared with other tissues. (C) All tissues frame-preservation gene numbers and (D) its corresponding delta-PSI.

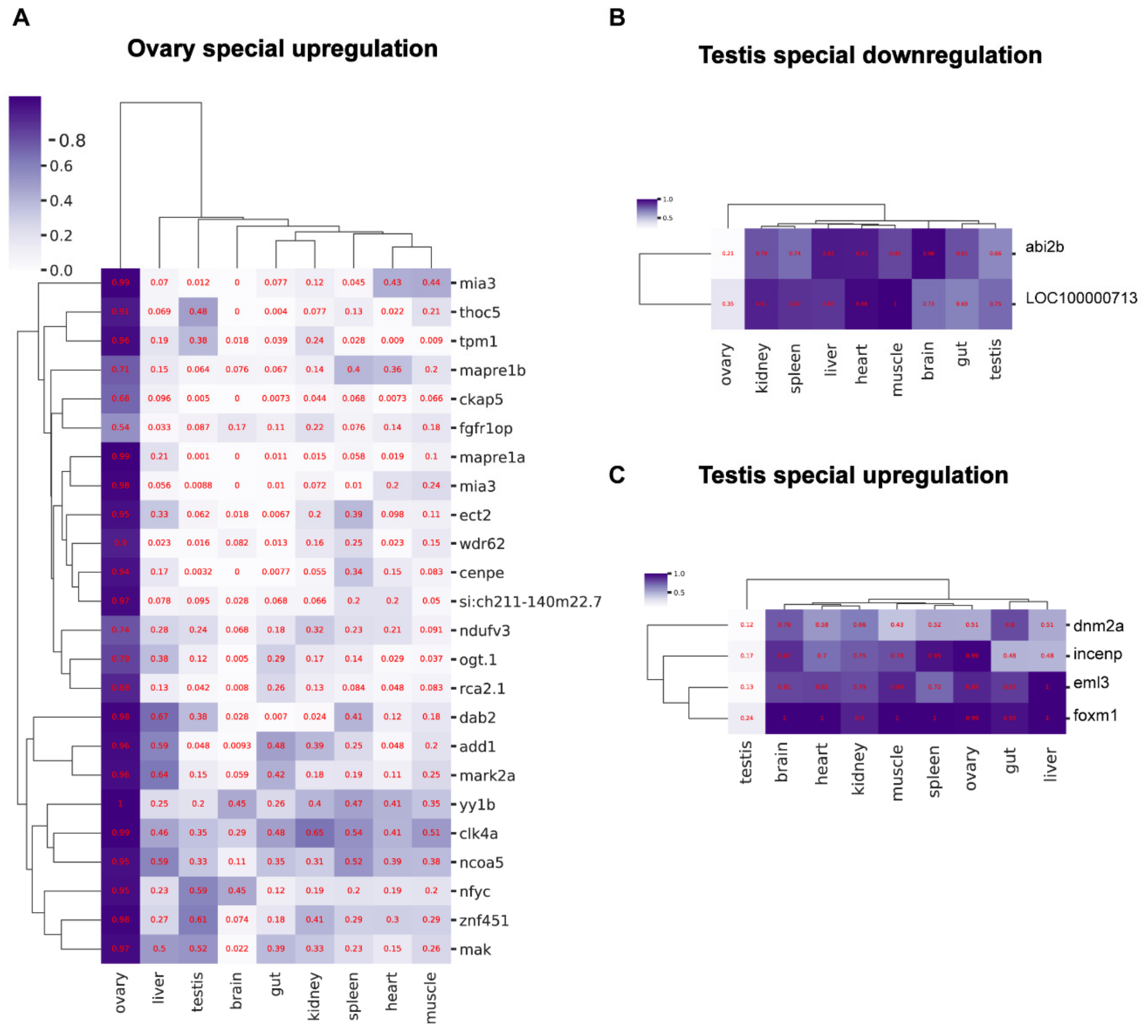

Figure S4: Pheatmap of potential testis- or ovary-specific DASGs. (A) pheatmap of ovary special upregulated DASGs. (B) pheatmap of testis special downregulated DASGs. (C) pheatmap of testis special upregulated DASGs.

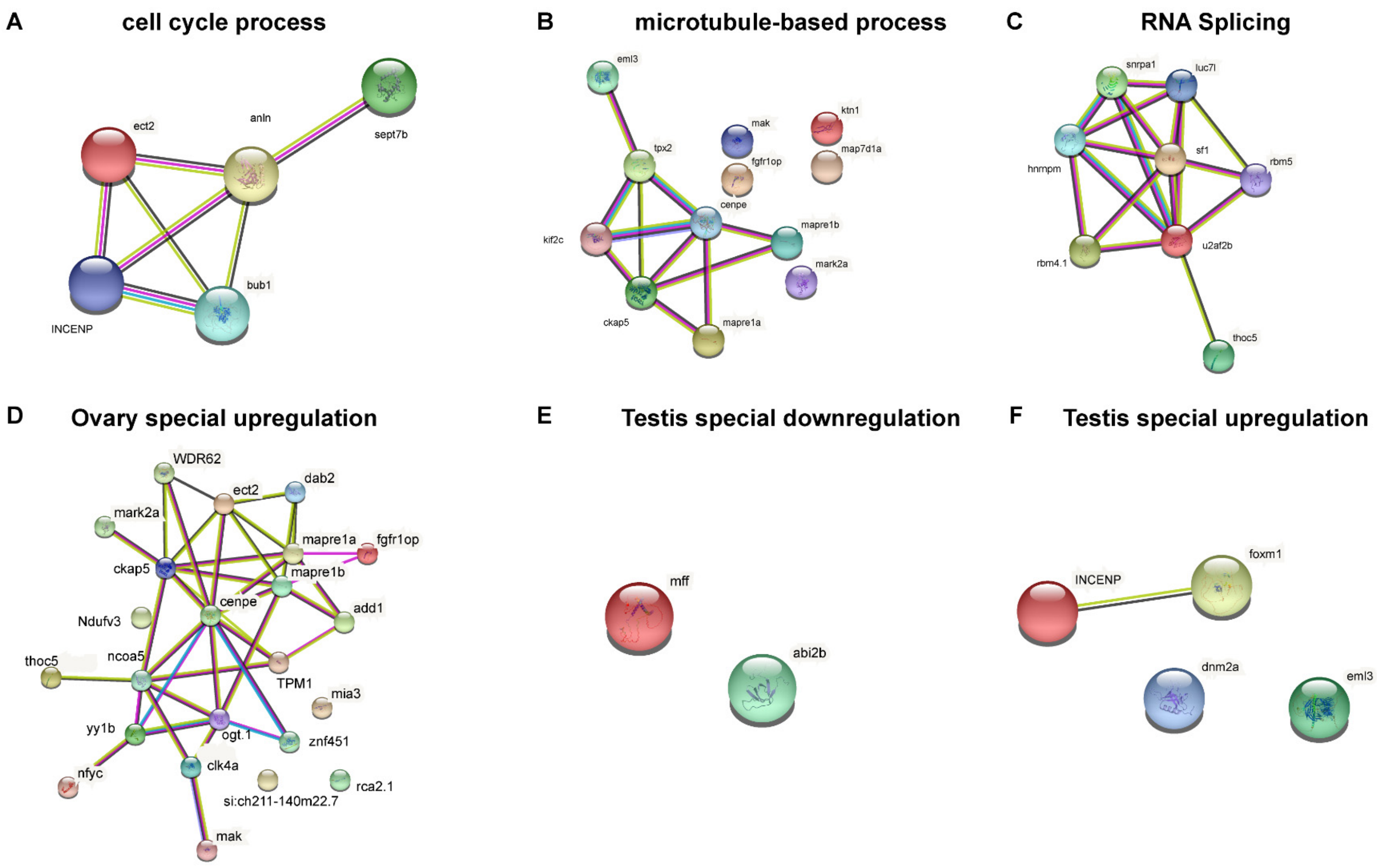

Figure S5: PPI networks's module analysis of potential testis- or ovary-specific DASGs.(A) PPI network of cell cycle process pathway DASGs.(B) PPI network of microtubule-based process pathway DASGs. (C) PPI network of RNA Splicing pathway DASGs. (D) PPI network of ovary special upregulated DASGs. (E) PPI network of testis special upregulated DASGs. (F) PPI network of testis special upregulated DASGs

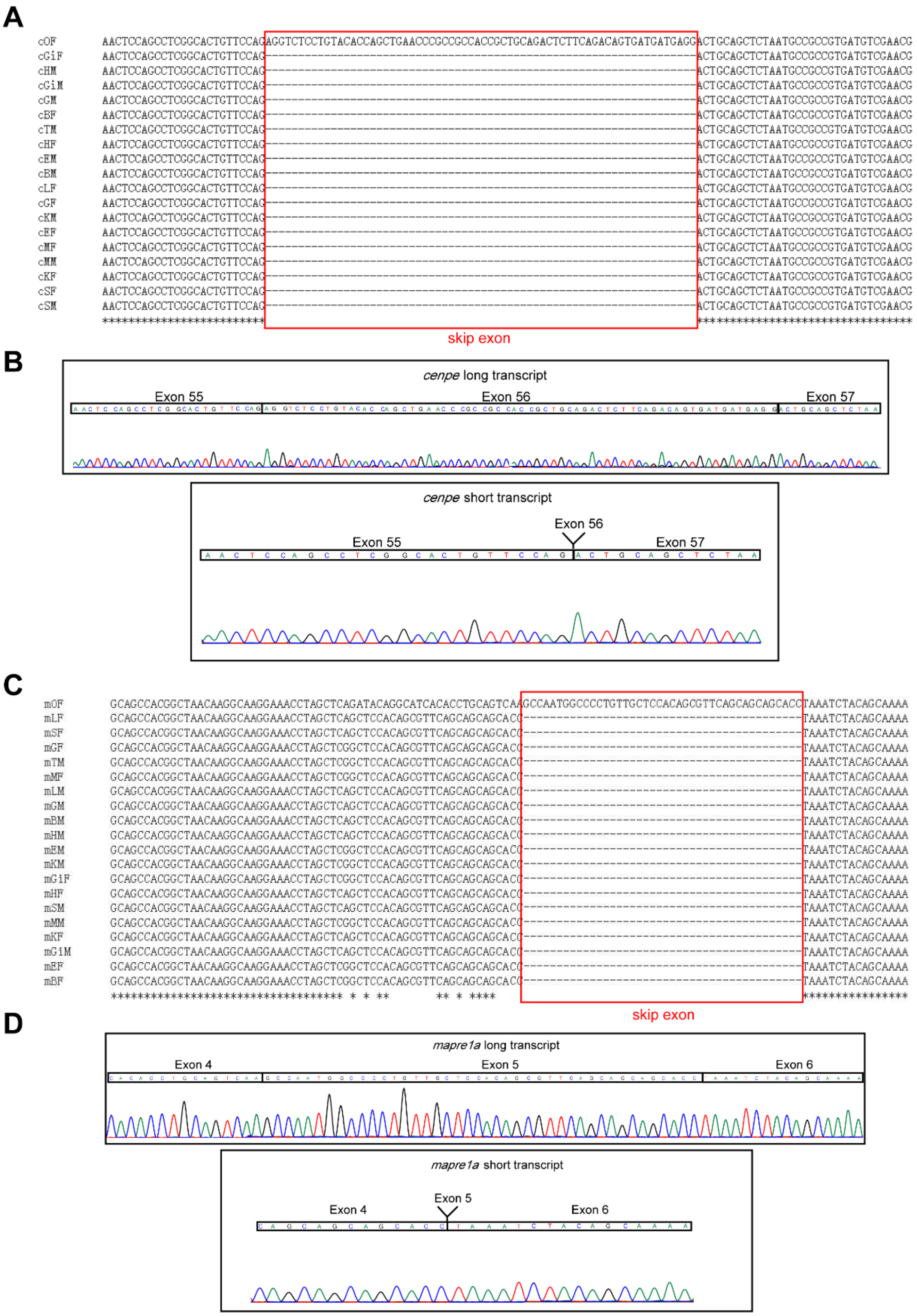

Figure S6: Sequencing data alignment and sequencing maps of long transcript and short transcript. (A) Sequencing data alignment of *cenpe*. (B) Sequencing maps of *cenpe* long transcript

and short transcript. (C) Sequencing data alignment of *mapre1a*. (D) Sequencing maps of *mapre1a* long transcript and short transcript. "cGiF": Female gill cenpe gene , "cEF": Female eye cenpe gene, "cBF": Female brain cenpe gene, "cHF": Female heart cenpe gene, "cLF": Female liver cenpe gene, "cSF": Female spleen cenpe gene, "cKF": Female kidney cenpe gene, "cGF": Female gut cenpe gene, "cMF": Female muscle cenpe gene, "cOF": Female ovary cenpe gene, "cGiM": Male gill cenpe gene, "cEM": Male eye cenpe gene, "cBM": Male brain cenpe gene, "cHM": Male heart cenpe gene, "cSM": Male spleen cenpe gene, "cKM": Male kidney cenpe gene, "cGM": Male gut cenpe gene, "cMM": Male muscle cenpe gene, "cTM": Male testis cenpe gene, "mGiF": Female gill mapre1a gene, "mEF": Female eye mapre1a gene, "mBF": Female brain mapre1a gene, "mHF": Female heart mapre1a gene, "mLF": Female liver mapre1a gene, "mLF": Female liver mapre1a gene, "mSF": Female spleen mapre1a gene, "mKF": Female kidney mapre1a gene, "mGF": Female gut mapre1a gene, "mMF": Female muscle mapre1a gene, "mOF": Female ovary mapre1a gene, "mGiM": Male gill mapre1a gene, "mEM": Male eye mapre1a gene, "mBM": Male brain mapre1a gene, "mHM": Male heart mapre1a gene, "mSM": Male spleen mapre1a gene, "mKM": Male kidney mapre1a gene, "mGM": Male gut mapre1a gene, "mMM": Male muscle mapre1a gene, "mTM": Male testis mapre1a gene.

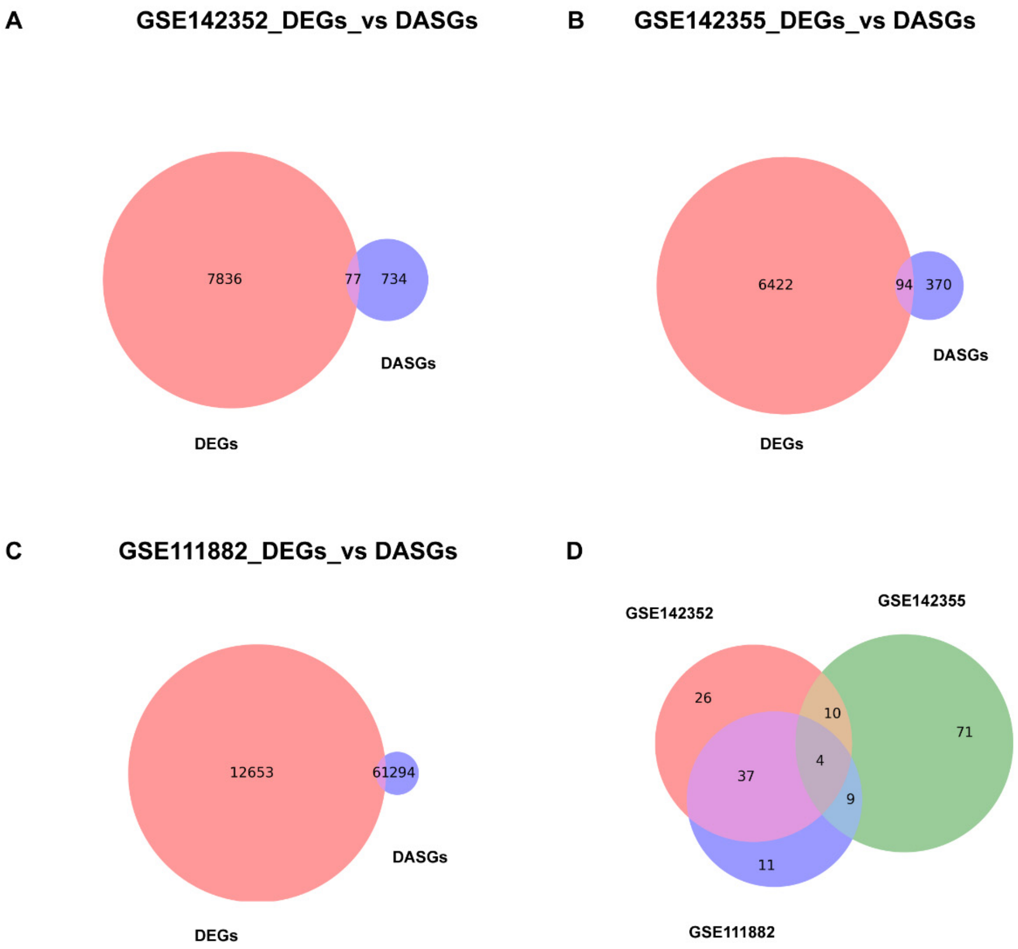

Figure S7: (A) overlap genes between DASGs and DEGs in GSE142352. (B) overlap genes between DASGs and DEGs in GSE142355. (C) overlap genes between DASGs and DEGs in GSE111882. (D) overlap genes between DASGs and DEGs shanred in three sets of dataset.
